# Respiratory syncytial virus infection provides protection against severe acute respiratory syndrome coronavirus challenge

**DOI:** 10.1128/jvi.00669-24

**Published:** 2024-08-28

**Authors:** Stacey M. Hartwig, Abby Odle, Lok-Yin Roy Wong, David K. Meyerholz, Stanley Perlman, Steven M. Varga

**Affiliations:** 1 Department of Microbiology and Immunology, University of Iowa, Iowa City, Iowa, USA; 2 Department of Pathology, University of Iowa, Iowa City, Iowa, USA; 3 Interdisciplinary Graduate Program in Immunology, University of Iowa, Iowa City, Iowa, USA; University of Kentucky College of Medicine, Lexington, Kentucky, USA

**Keywords:** respiratory syncytial virus, lung infection, SARS-CoV-2

## Abstract

Respiratory infections are a major health burden worldwide. Respiratory syncytial virus (RSV) is among the leading causes of hospitalization in both young children and older adults. The onset of the severe acute respiratory syndrome coronavirus 2 (SARS-CoV-2) pandemic and the public health response had a profound impact on the normal seasonal outbreaks of other respiratory viruses. However, little is known about how a prior respiratory virus infection impacts SARS-CoV-2 disease outcomes. In this study, we examine the impact of a previous RSV infection on the disease severity of a subsequent SARS-CoV-2 challenge in BALB/c mice. Mice infected with RSV, followed by a SARS-CoV-2 challenge, 30 days later, exhibited decreased weight loss and increased survival as compared to control groups. Our results suggest a prior RSV infection can provide protection against a subsequent SARS-CoV-2 infection.

**IMPORTANCE:**

Severe acute respiratory syndrome coronavirus 2 and respiratory syncytial virus are respiratory viruses that are a major health burden worldwide. Severe acute respiratory syndrome coronavirus 2 and respiratory syncytial virus frequently have peak seasonal outbreaks during the winter months, and are capable of causing severe respiratory disease, often leading to hospitalization. The 2019 pandemic brought attention to the importance of understanding how co-circulating viruses can impact the disease severity of other respiratory viruses. It is known that many hospitalized patients are undergoing multiple viral infections at once, yet not much has been studied to understand the impact this has on other respiratory viruses or patients. How co-circulating viruses impact one another can provide critical knowledge for future interventions of hospitalized patients and potential vaccination strategies.

## INTRODUCTION

In 2019, the arrival of severe acute respiratory syndrome coronavirus 2 (SARS-CoV-2) rapidly caused a pandemic and a major healthcare crisis. Before the pandemic, respiratory tract infections were already a major health burden worldwide, and the onset of the pandemic exacerbated this burden on healthcare systems. Over 750 million confirmed cases of SARS-CoV-2 and over 6 million deaths have been reported by the World Health Organization as of September 2023 (https://www.who.int/emergencies/diseases/novel-coronavirus-2019). It is common that many respiratory viruses co-circulate, and individuals are likely exposed to multiple single respiratory viral infections each year. Viral coinfections are detected in as many as 20%–30% of hospitalized individuals. While some coinfection combinations result in reduced pathogenicity, others severely worsen disease (1). Furthermore, the impact of previous respiratory viral infection on subsequent viral infections, in particular SARS-CoV-2, is understudied.

Respiratory syncytial virus (RSV) is among the leading cause of morbidity and mortality in young children as well as the elderly population (2). Normal RSV seasonal outbreaks typically occur during the winter months in the United States and have overlapping seasons with other respiratory viruses (3). With the onset of the SARS-CoV-2 pandemic, RSV and other respiratory pathogen infections were remarkably decreased in the winter months following the initial outbreak of the SARS-CoV-2 virus. Most of the decrease in cases can potentially be explained by the public health interventions instituted around the world (4–6). However, as these restrictions eased, a resurgence in infections by other respiratory pathogens was observed (7). Specifically in 2021, RSV cases increased during spring and early summer months. The 2022 RSV seasonal outbreak began later than the previous year; however, it was still earlier than pre-pandemic cycles, and an increase in the peak of positive cases was observed (8).

The SARS-CoV-2 pandemic has brought attention to how co-circulating viruses can impact one another. Previous studies have shown that a prior or simultaneous infection with influenza A virus (IAV) exacerbates SARS-CoV-2 infection (9–12). However, much less is known regarding the impact of RSV and SARS-CoV-2 coinfections. We have previously reported that RSV infection protects against a subsequent IAV infection, thereby demonstrating the protective capacity of RSV against an unrelated respiratory virus (13). In this study, we demonstrate that prior infection with RSV also profoundly alters the morbidity and mortality of a subsequent SARS-CoV-2 infection.

## RESULTS

### Prior RSV infection influences morbidity and mortality of a subsequent SARS-CoV-2 infection

**FIG 1:**
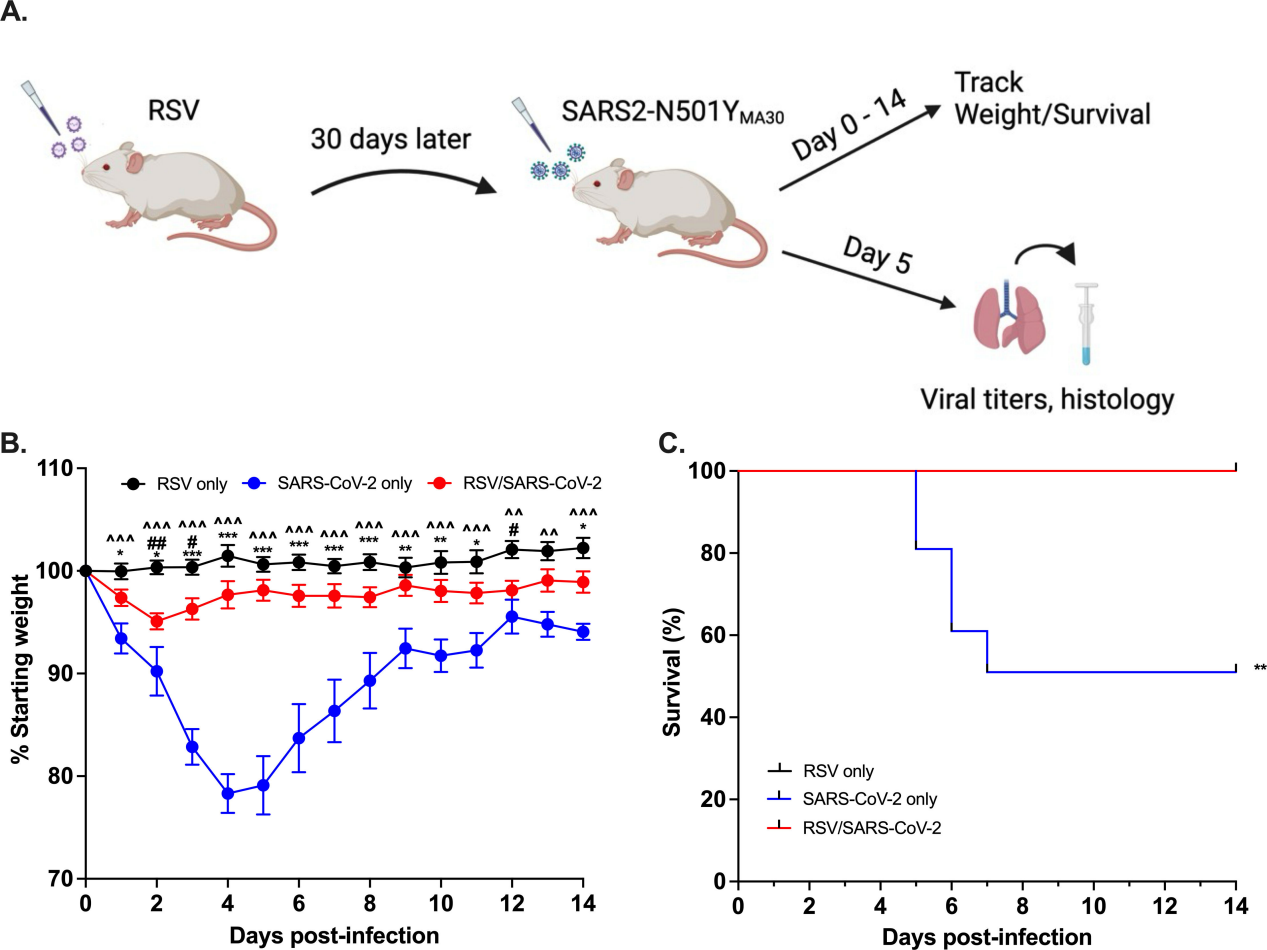
RSV coinfection confers protection in SARS-CoV-2-N501Y_MA30_-infected BALB/c mice. Schematic design of experimental design (A). Female 6–8 week-old BALB/c mice were infected i.n. with 2–4.8 × 10^6^ PFU RSV and 30 days later challenged with 800 PFU of SARS-CoV-2. Mice were monitored daily for weight loss (B) and survival (C). Black (RSV only) were initially infected with RSV and received PBS 30 days post RSV infection. Blue (SARS-CoV-2 only) initially received PBS and were infected with SARS-CoV-2 30 days later. Red (RSV/SARS-CoV-2) were initially infected with RSV and were subsequently infected with SARS-CoV-2 30 days later. Data were combined from two independent experiments, *n* = 10 for each group. Data in (B) were analyzed using a two-way ANOVA with a Tukey post-hoc test. Data in (C) were analyzed using a Mantel–Cox test. Comparisons are ^RSV only to SARS-CoV-2 only; #RSV only to RSV/SARS-CoV-2 and *SARS-CoV-2 only to RSV/SARS-CoV-2. ^*P* < 0.05*, ^^P* < 0.01*, ^^^P* < 0.001; #*P* < 0.05*, ##P* < 0.01; *P < 0.05, **P < 0.01, ***P < 0.001. Diagram created using BioRender.com.

We sought to determine the effects of a prior RSV infection on a subsequent SARS-CoV-2 infection. Mice were infected with RSV or phosphate-buffered saline (PBS) on day 0 and 30 days later were challenged with either 800 pfu or 5,000 pfu of mouse-adapted SARS-CoV-2-N501Y_MA30_ or PBS and assessed for weight loss, survival, lung titers, and histology (Fig. 1A). Mice were also infected with each single virus as controls. Following infection with the lower dose of SARS-CoV-2-N501Y_MA30_, all groups of mice were monitored daily. Mice infected with RSV followed by SARS-CoV-2-N501Y_MA30_ exhibited significantly less weight loss as compared to that infected with SARS-CoV-2-N501Y_MA30_ only (Fig. 1B). Mice infected with RSV followed by SARS-CoV-2-N501Y_MA30_ also demonstrated a significant reduction in mortality, with no mice succumbing to illness, as compared to mice infected with SARS-CoV-2-N501Y_MA30_ only, in which 50% of mice succumbed by day 7 post-infection (p.i.) (Fig. 1C). These data demonstrate that a prior infection of RSV can prevent disease severity of SARS-CoV-2-N501Y_MA30_.

### Prior RSV infection reduces SARS-CoV-2 viral load

**FIG 2:**
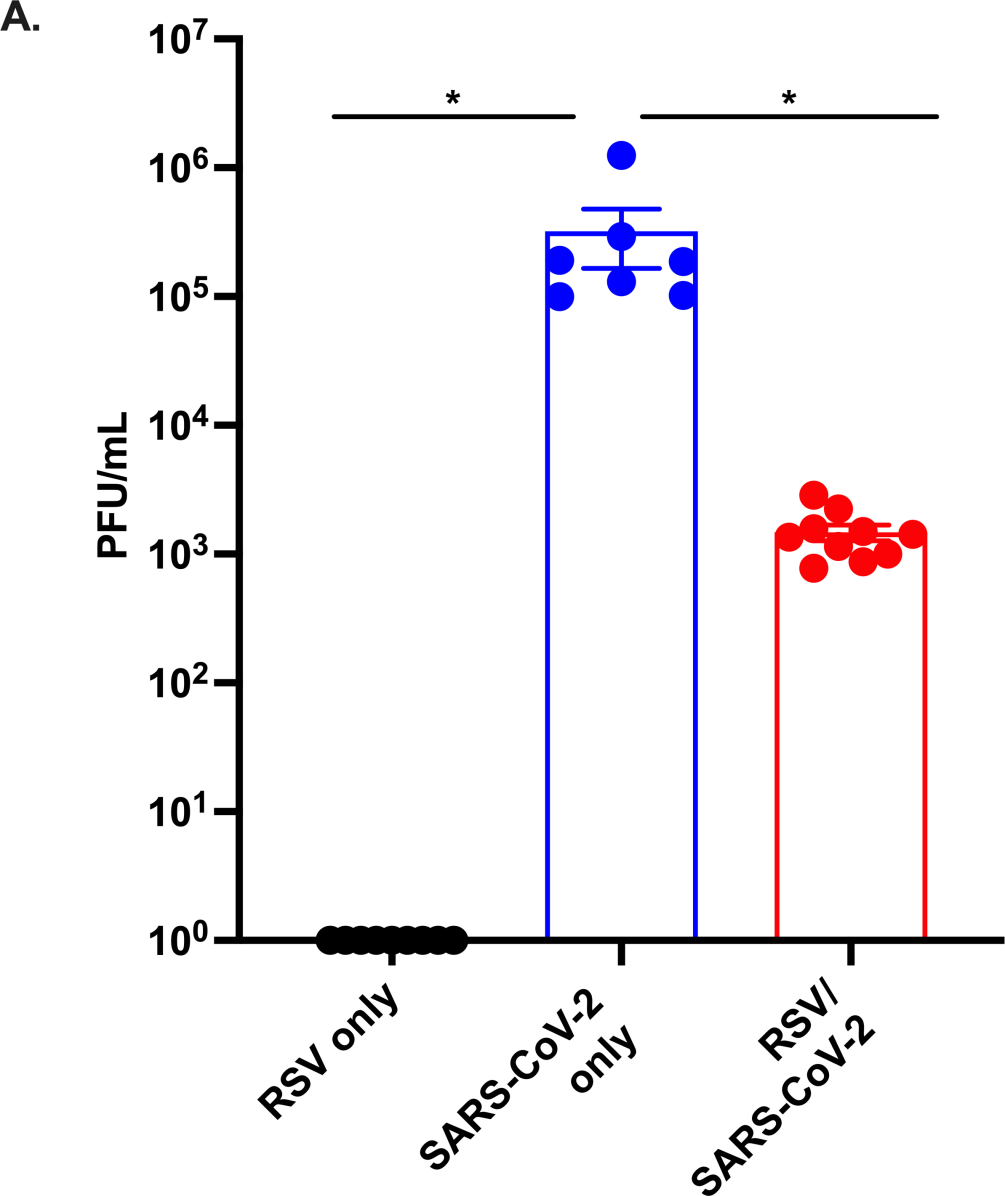
Prior RSV infection reduces viral titers in mice infected with a lethal SARS-CoV-2-N501Y_MA30_. Female 6–8 week-old BALB/c mice were infected i.n. with 2–4.8 × 10^6^ PFU RSV and 30 days later challenged with a lethal dose, 5,000 PFU of SARS-CoV-2. Lungs were harvested from mice at 5 days post SARS-CoV-2 infection. RSV/SARS-CoV-2 mice exhibited reduced lung titers compared to SARS-CoV-2-only group (A). Data were combined from two independent experiments, *n* = 10/group. Data were analyzed using a one-way ANOVA with Tukey post-hoc test. *P < 0.05.

Having observed reduced morbidity and mortality in co-infected mice, we next determined the impact of a prior RSV infection on SARS-CoV-2-N501Y_MA30_ viral burden. Mice were infected with either RSV or PBS on day 0 and 30 days later challenged with a lethal dose of SARS-CoV-2-N501Y_MA30_ or PBS. On day 5 post-SARS-CoV-2-N501Y_MA30_ infection, mice infected with RSV followed by SARS-CoV-2-N501Y_MA30_ showed significant reduction in viral titers as compared to the SARS-CoV-2-N501Y_MA30_ only group (Fig. 2).

### Prior RSV infection diminishes pathological changes in SARS-CoV-2 infected mice

**FIG 3:**
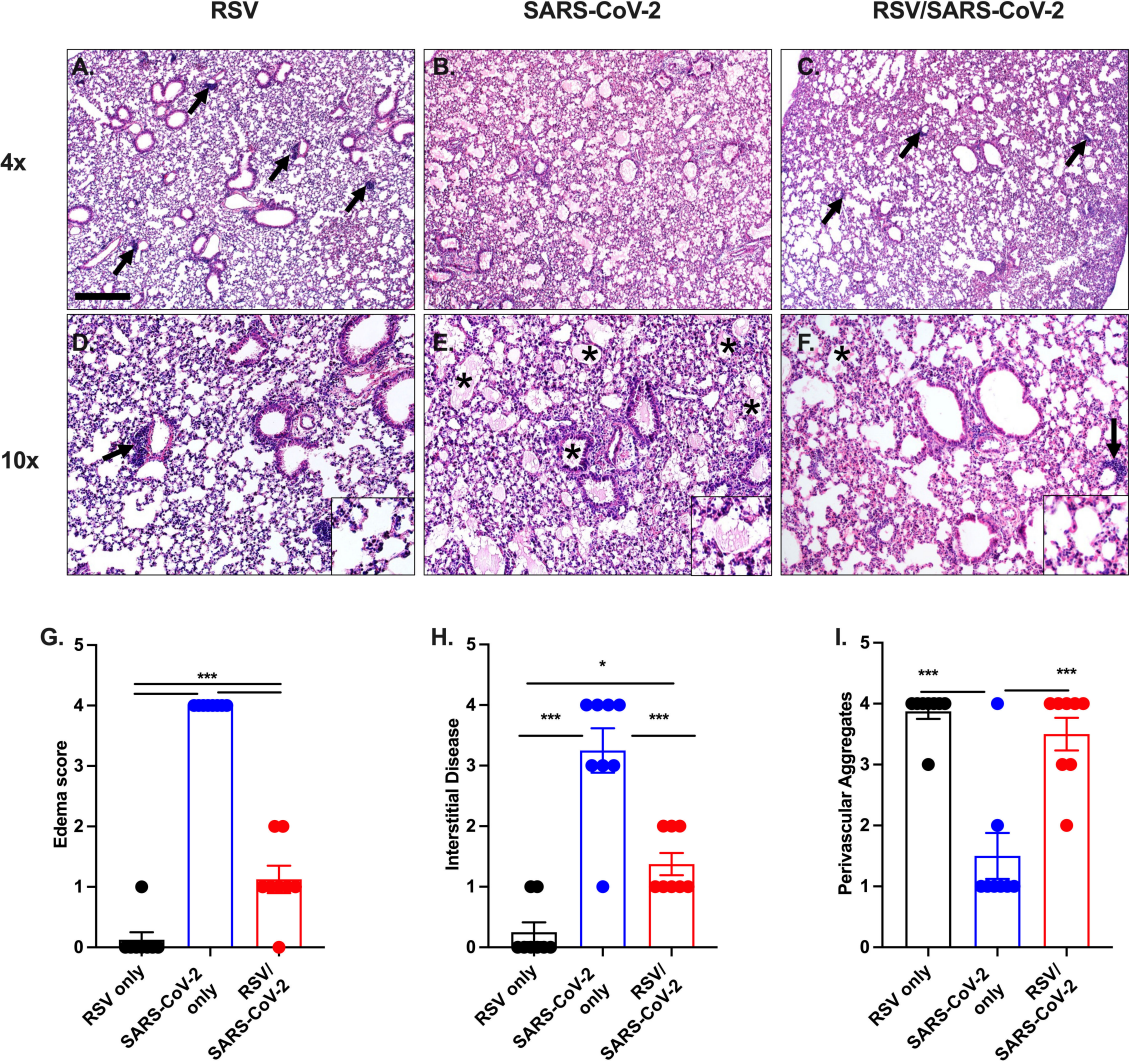
Prior RSV infection diminishes pathological changes in mice infected with a lethal SARS-CoV-2-N501Y_MA30_. Lungs were harvested from mice at 5 days post SARS-CoV-2 infection. RSV-only infected lungs exhibited focal lymphoid aggregates (arrows, iBALT-like) (A and D), SARS-CoV-2 lungs had extensive edema (B and E) (*) and RSV/SARS-CoV-2 group had rare edema (C and F). Bar = 435 and 175 µm. Overall edema (G) and interstitial disease (H) scoring showed a significant reduction in lung pathology of co-infected mice, while overall perivascular aggregates (I) scoring showed a significant similar pathology as the RSV-only group. Data were combined from two independent experiments, *n* = 10/group. Data in (G, H, and I) were analyzed using a one-way ANOVA with Tukey post-hoc test. *P < 0.05; ***P < 0.001.

Having observed reduced weight loss and viral titers, leading to increased survival, we next assessed lung pathology of RSV/SARS-CoV-2-N501Y_MA30_ infected mice. RSV-only infected mice exhibited inducible bronchiolar-associated lymphoid tissue (iBALT)-like focal lymphoid aggregates in the lungs 35 days post-RSV infection (Fig. 3A and D). SARS-CoV-2-N501Y_MA30_ only infected mice exhibited extensive edema 5 days p.i. as compared to other groups (Fig. 3B and E). RSV/SARS-CoV-2-N501Y_MA30_ infected mice exhibited significantly reduced lung pathology as compared to the SARS-CoV-2-N501Y_MA30_ single virus-only control (Fig. 3C and F). Reduced lung pathology was prominently demonstrated through the analysis of alveolar edema (Fig. 3G), which is a histopathological marker of SARS-CoV-2 disease severity (e.g., lethality) in mice and in the reduced distribution of interstitial disease (Fig. 3H), which normally consists of a more widespread neutrophilic mixed cellularity distribution (14). However, RSV-only and co-infected mice demonstrated similar perivascular lymphoid aggregates in the lungs as compared to the SARS-CoV-2-only group (Fig. 3I). These data taken together demonstrate that a prior RSV infection confers protection from SARS-CoV-2-N501Y_MA30_-induced disease severity, even when challenged with a lethal dose of SARS-CoV-2-N501Y_MA30_.

## DISCUSSION

Since the onset of the ongoing global SARS-CoV-2 pandemic, the study of both overlapping and sequential viral infections has become increasingly important. We have previously reported that prior infection with RSV ameliorates IAV-induced disease (13). Here, we sought to determine whether RSV would diminish SARS-CoV-2-induced disease. Similar to our previous findings with RSV/IAV sequential infections, we demonstrate that mice previously infected with RSV followed by SARS-CoV-2-N501Y_MA30_ challenge 30 days later, significantly reduced weight loss, viral titers, and lung pathology. Survival rate was also significantly increased in mice previously infected with RSV as compared to SARS-CoV-2-N501Y_MA30_ single virus control. Our data suggest that a prior RSV infection significantly reduces the replication of SARS-CoV-2-N501Y_MA30_ in the lung.

Most previous SARS-CoV-2 co-viral infection studies have focused on influenza and SARS-CoV-2. However, several of these studies have reported conflicting results. Studies performed by Oishi et al. demonstrated that hamsters infected with SARS-CoV-2 and IAV simultaneously, displayed a reduction in viral titers of SARS-CoV-2 at days 3 and 5 p.i., but no reduction of IAV titers was observed. When hamsters were previously infected with IAV and challenged with SARS-CoV-2, 3, 7, or 14 days later SARS-CoV-2 titers were significantly reduced early p.i., and IAV titers were unaffected (15). In contrast, Bai et al. demonstrated that a prior IAV infection followed by a SARS-CoV-2 challenge enhanced SARS-CoV-2 viral replication (16). Differences in animal models, timing of coinfection, and viral strains used could explain the discrepancy in the results between both groups and our data.

In a study performed by Achdout et al., IAV/SARS-CoV-2 coinfection exacerbated IAV viral loads in the lung and nasal turbinates but resulted in a reduction of SARS-CoV-2 viral load (17). When examining the impact of IAV vaccination on subsequent SARS-CoV-2 single infection, the authors observed no difference in survival compared to unvaccinated mice infected with SARS-CoV-2. However, IAV vaccination was shown to provide significant protection against subsequent IAV/SARS-CoV-2 coinfection challenge. In contrast, immunization with SARS-CoV-2 did not show any protection against IAV/SARS-CoV-2 coinfection challenge (17). Similarly, Huang et al. also demonstrated in ferrets a seasonal IAV vaccine ameliorated disease severity caused by IAV and SARS-CoV-2 coinfection as compared to nonvaccinated animals (18).

Many of the published coinfection studies, including the studies discussed above, examined overlapping infections. In contrast, our study examined the impact of successive RSV and SARS-CoV-2 infections that are spaced 30 days apart. The 30-day spacing ensures that RSV has been cleared from the lungs of mice, in addition to the cessation of the innate and adaptive response to active RSV infection. Our studies with IAV demonstrate that RSV-mediated protection is independent of both B and T cells (13). Additional studies will need to be performed to determine the cell types contributing to RSV-mediated protection against SARS-CoV-2.

Regardless of whether findings have shown that coinfections have beneficial or worsening effects, co-viral infection models have become increasingly important to study. Understanding how co-circulating viruses impact one another can provide important information not only for patient testing and treatment regimens of hospitalized patients but also for understanding the importance of seasonal vaccines and improving vaccine design as well.

## MATERIALS AND METHODS

### Mice

Female BALB/c mice 6–8 weeks of age were obtained from the National Cancer Institute (NCI, Fredrick, MD).

### Virus growth and mice infection

RSV A2 strain was propagated in HEp-2 cells (American Type Culture Collection) as previously described (19). Mice were infected intranasally (i.n.) with 2–4.8 × 10^6^ PFU RSV, while under light anesthesia with isoflurane. Mouse-adapted SARS-CoV-2N501Y_MA30_ was propagated in Calu3 cells as previously described (20). Thirty days later, mice were anesthetized with ketamine-xylazine and i.n. infected with either 800 PFU or 5,000 PFU of SARS-CoV-2-N501Y_MA30_ in a total volume of 50 µL of Dulbecco’s Modified Eagle Medium (DMEM). Mice were monitored daily for morbidity (weight loss) and mortality for up to 14 days postinfection during both the RSV and SARS-CoV-2-N501Y_MA30_ infections. Mice infected with a lethal dose of SARS-CoV-2-N501Y_MA30_ were sacrificed at day 5 postinfection and lungs were harvested to determine viral titers and for histopathological examination. All work with SARS-CoV-2 was conducted in the University of Iowa Biosafety Level 3 (BSL-3) Laboratory.

### Viral titers

Lungs were harvested at 5 days postinfection and homogenized in 1 mL PBS, aliquoted into micro tubes, and kept at −80°C. Lung homogenates were then serially diluted in DMEM and used to inoculate Vero E6 cells in 12 well plates followed by incubation at 37°C in 5% CO_2_ for 1 h with gentle rocking every 10–15 min. After removing the inoculum, plates were overlaid with 1.2% agarose containing 2% fetal bovine serum (FBS). After further incubation for 3 days, overlays were removed, and plaques were visualized by staining with 0.1% crystal violet. Viral titers were calculated as PFU per milliliter.

### Histopathology

Lungs were perfused with 5–10 mL of PBS, then harvested, and fixed with zinc formalin. Fixed tissues were embedded in paraffin, sectioned (~4 µm), and stained with hematoxylin and eosin. These tissues were examined by a boarded veterinary pathologist experienced with the model and scored using the post-examination method of masking the pathologist to group assignment (21). Edema distribution in the lung was ordinally scored: 0, none; 1, <25%; 2, 26%–50%; 3, 51%–75%; and 4, >75% of 200× magnification tissue fields (20). Interstitial disease with neutrophilic cellularity was similarly scored on a distribution basis. Perivascular lymphoid aggregates were ordinally scored as previously described (22). High-resolution images were taken using a BX53 microscope, DP73 digital camera, and Cell Sens Dimension software (Olympus).

### Statistics

All statistical analyses were performed using Prism software (GraphPad Software, Inc., San Diego, CA, USA). Statistical significance was determined by either one-way or two-way ANOVA with a Tukey post-hoc test and a Mantel–Cox test where designated. A value of *P* < 0.05 was considered significant. Asterisks indicating significance were defined as follows: *P < 0.05; **P < 0.01; ***P < 0.001 and when more than one comparison is being made #*P* < 0.05; ##*P* < 0.01; ###*P* < 0.001; ^*P* < 0.05; *^^P* < 0.01, *^^^P* < 0.001 are also used.

## Data Availability

All data are available from the corresponding author upon request.
